# DOTA-Derivatives of Octreotide Dicarba-Analogs with High Affinity for Somatostatin sst_2,5_ Receptors

**DOI:** 10.3389/fchem.2017.00008

**Published:** 2017-02-23

**Authors:** Alessandro Pratesi, Mauro Ginanneschi, Marco Lumini, Anna M. Papini, Ettore Novellino, Diego Brancaccio, Alfonso Carotenuto

**Affiliations:** ^1^Department of Chemistry “Ugo Schiff,” University of FlorenceFirenze, Italy; ^2^Interdepartmental Laboratory of Peptide & Protein Chemistry & Biology, University of FlorenceFirenze, Italy; ^3^Department of Pharmacy, University of Naples “Federico II”Naples, Italy

**Keywords:** somatostatin receptors, dicarba-analogs, DOTA-conjugation, NMR conformational analysis, radiotracers

## Abstract

*In vivo* somatostatin receptor scintigraphy is a valuable method for the visualization of human endocrine tumors and their metastases. In fact, peptide ligands of somatostatin receptors (sst's) conjugated with chelating agents are in clinical use. We have recently developed octreotide dicarba-analogs, which show interesting binding profiles at sst's. In this context, it was mandatory to explore the possibility that our analogs could maintain their activity also upon conjugation with DOTA. In this paper, we report and discuss the synthesis, binding affinity and conformational preferences of three DOTA-conjugated dicarba-analogs of octreotide. Interestingly, two conjugated analogs exhibited nanomolar affinities on sst_2_ and sst_5_ somatostatin receptor subtypes.

## Introduction

**Figure 1:**
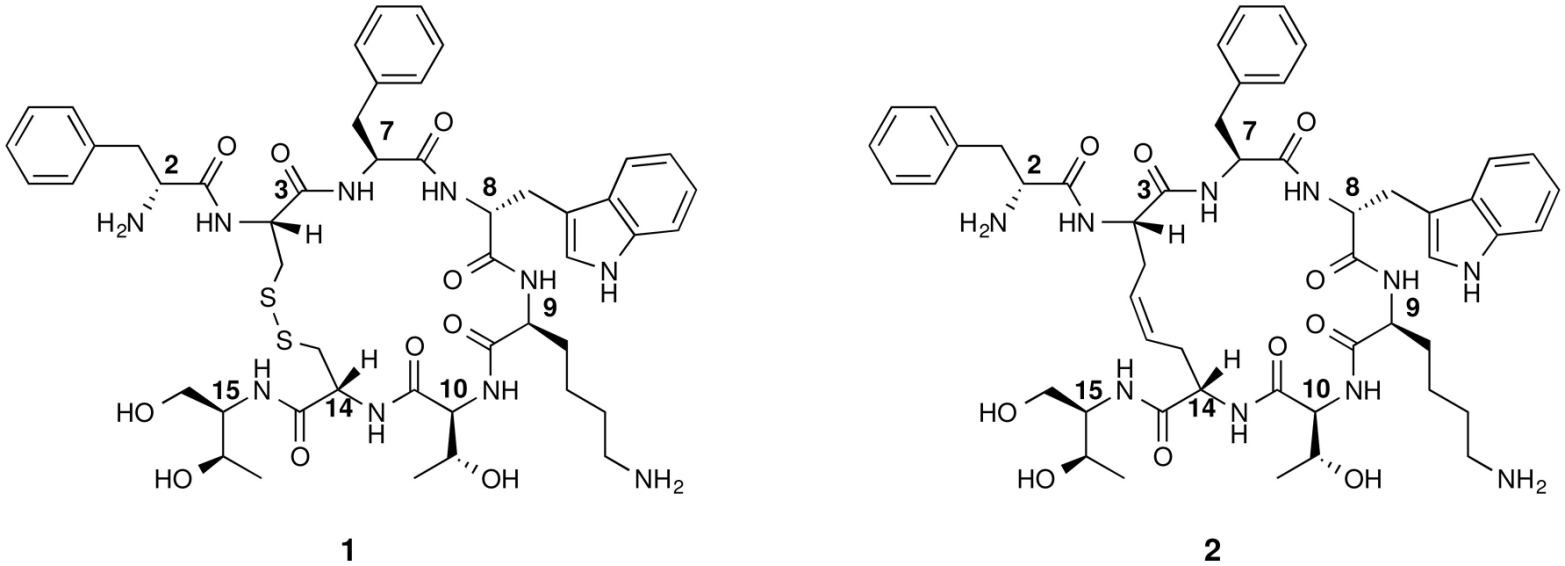
**Chemical structure of octreotide (1) and of the first dicarba-tethered analog (2)**. Numbering of the amino acid residues follows that of the native SRIF.

Radiolabeled peptides, targeting specific receptors of malignant cells, *in primis* GPCRs, have emerged in the past years as one of the most promising tools for diagnosis and therapy of several kinds of metastatic tumors which express these receptors (Ramogida and Orvig, 2013). Somatostatins SRIF-14 and SRIF-28 are peptide hormones, which have a wide range of pharmacological actions on exocrine and endocrine secretions (Brazeau et al., 1973) mediated by direct interaction with at least five GPCRs. Chemically, they are -S-S- bridged cyclic peptides containing 14 and 28 amino acids, respectively. SRIF receptors are highly expressed in various types of malignant cells, particularly in some neuroendocrine tumors (NETs) or neuroendocrine-like diseases. The use of native SRIF-14, that possesses strong antisecretive and antiproliferative properties, was hampered by the short *in vivo* half-life of this hormone (<3 min; Weckbecker et al., 2003). This prompted several research groups, during the last three decades, to synthetize a huge number of new size-reduced cyclic analogs with enhanced stability in physiological conditions. Despite this synthetic effort, only a few analogs resulted as good candidates for further *in vitro* and *in vivo* investigations and fewer entered the clinical practice. Among them, the cyclo-octapeptide octreotide (Figure 1) emerged as sst_2_ agonist, thanks to the high affinity and specificity toward this receptor that is over-expressed by numerous NETs. However, the octreotide itself is a poor inhibitor of the cell growth and it is currently used as a carrier of radionuclides for diagnostic and therapeutic purposes. Therefore, octreotide and its congeners like [Tyr^3^]-octreotide (TOC), [1-Nal^3^]-octreotide (NOC) or [Tyr^3^, Thr^8^]-octreotide (TATE), conjugated with radiolabelled chelating agents like HYNIC, DTPA, or DOTA, gave good results when applied in imaging and therapy of NETs (Ginj et al., 2006; Ambrosini et al., 2011; Graham and Menda, 2011; Maecke and Reubi, 2011).

**Figure 2:**
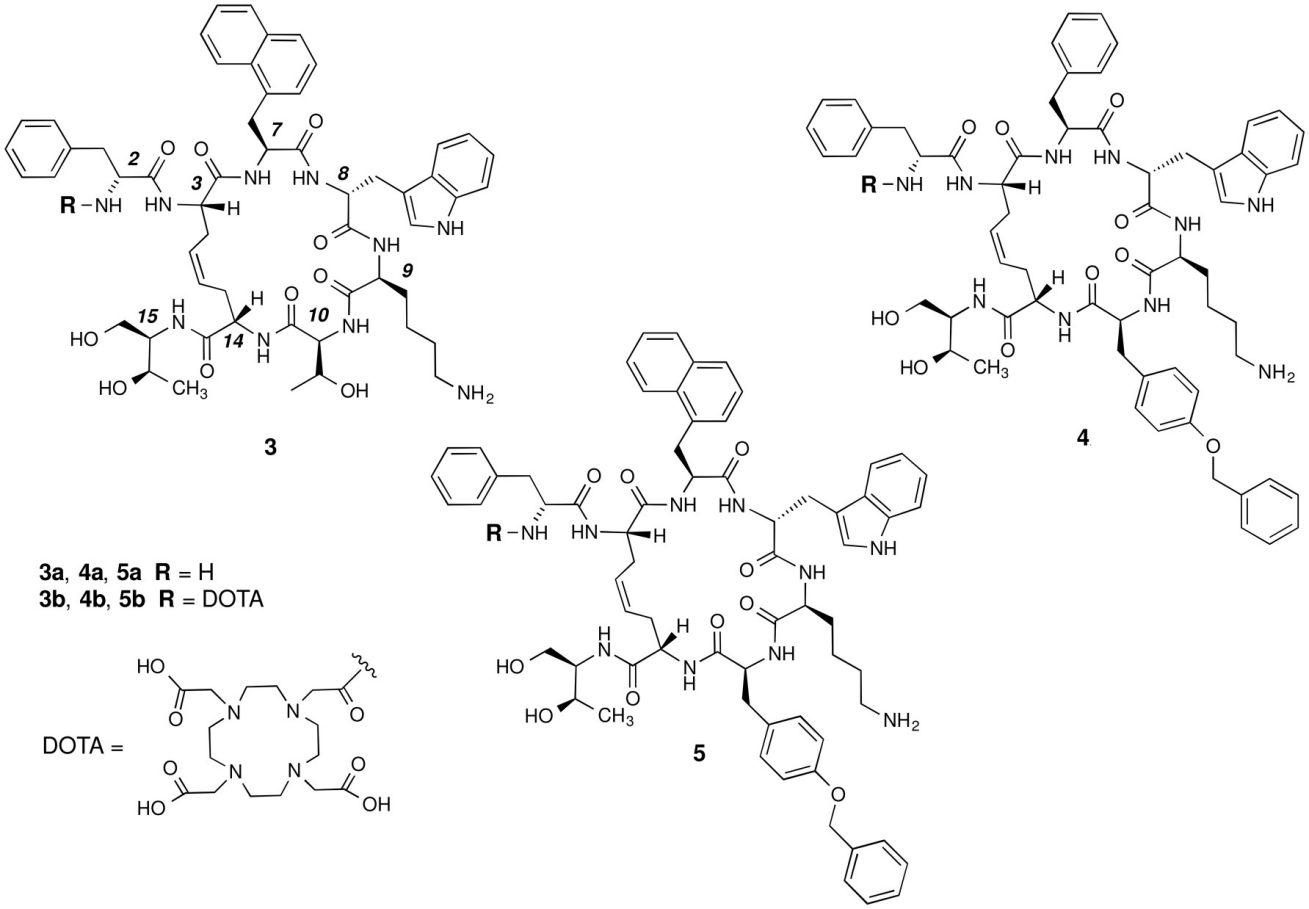
**Chemical structures of the studied dicarba-analogs of octreotide**. Numbering of the amino acid residues follows that of the native SRIF.

The basic parameters needed for satisfactory clinical applications of radiolabeled SRIF analogs have been recently pointed out by Maecke and Reubi (2011). During the last three decades, Rivier's group carried out an impressive work by synthesizing a tremendous number of SRIF analogs, agonists and antagonists, and correlating their affinity to sst_1−4_ receptors with their conformation in solution (Grace et al., 2008). However, all these synthetic cyclopeptides were bridged by S-S clasp, mimicking the native SRIF tether, that is sensitive to enzymatic and chemical agents. This prompted us to prepare more robust octreotide dicarba-analogs by RCM between the allylic side chains of two l-allylglycine (Agl) residues (Agl^3^ and Agl^14^) of the linear peptides. The structures of octreotide (**1**) and of the first dicarba-tethered analog prepared in our laboratories (**2**) (Carotenuto et al., 2005) are reported in Figure 1. Despite a very similar amino acid sequence, the affinity of **2** toward the sst_2_ receptor was about 70-fold less than that reported for compound **1** (D'Addona et al., 2008). No affinity improvement was detected increasing the flexibility of **2** by double bond hydrogenation. Later on, we prepared several other dicarba-analogs by changing selected amino acids in the sequence of the analog **2**. Some of the novel compounds showed affinity for sst_2,3,5_ subtypes in the nM range (D'Addona et al., 2008; Di Cianni et al., 2010). Moreover, our studies on the conformation-affinity relationship revealed that the cyclic dicarba-analogs, having high affinity for the sst_5_ subtype, showed propensity to form a 3_10_-helix at the C-terminal sequence (Di Cianni et al., 2010). The structures of these compounds are reported in Figure 2.

In this paper, we describe the conjugation of the dicarba- analogs **3a**, **4a**, and **5a** with the DOTA chelating agent, thus obtaining the novel cyclic peptides **3b**, **4b**, and **5b**, which were tested for their affinity toward sst_2_ and sst_5_ receptors. As expected, the introduction of the DOTA moiety at the *N*-terminus of the cyclic peptides affected the receptors binding affinity of these molecules (Table 1). We here report and discuss on the synthesis, the receptor binding affinity and conformational preferences of these novel DOTA-conjugated octreotide dicarba-analogs.

**Table 1:** **Sst_2,5_ receptor affinities of the free and DOTA-conjugates octreotide dicarba-analogs**.

**Compound**	**Receptor (IC_50_, nM)**	
	**sst_2_**	**sst_5_**
3a^a^	9.6 ± 0.9	16.5 ± 4.5
3b	61 ± 5.4	130 ± 7
4a^a^	46 ± 3	12.3 ± 0.3
4b	>300	91 ± 7.8
5a^a^	101 ± 9	4.9 ± 1.0
5b	160 ± 8	12 ± 2.6

*The mean values ± SEM were obtained on the bases of three independent repetitions of the experiment. a Data published previously (Di Cianni et al., 2010)*.

## Materials and methods

### General procedures

Fmoc protected amino acids were purchased from Novabiochem (Laufelfingen, Switzerland) and from Iris Biotech (Marktredwitz, Germany), H-l-Thr(*t*Bu)-ol-2-chlorotrityl resin and HATU were purchased from Iris Biotech. Second generation Grubbs catalyst was obtained from Aldrich (St. Louis, MO, USA). Fmoc-Agl-OH was purchased from Polypeptide Laboratories (Strasbourg, France). Peptide grade DMF was from Scharlau (Barcelona, Spain). TSP was purchased from MSD Isotopes (Montreal, Canada). ^2^H_2_O was obtained from Aldrich. SDS-d_25_ was obtained from Cambridge Isotope Laboratories, Inc. (Andover, MA). All the other solvents and reagents used for SPPS were of analytical quality and used without further purification. The solid phase syntheses and DOTA-conjugations were performed using a semi-automatic synthesizer from MultiSynTech (Witten, Germany). Analytical RP-HPLCs were performed on a Waters instrument equipped with a UV detector on a Phenomenex Jupiter C18 column (5 μm, 250 × 4.6 mm) using a flow rate of 1 mL/min, with the following solvent system: 0.1% TFA in H_2_O (A), 0.1% TFA in MeCN (B). Semi-preparative RP-HPLC were performed on the same instrument using a flow rate of 4 ml/min with the same solvent system, on a Phenomenex Jupiter C18 column (10 μm, 250 × 10 mm). Mass spectra were registered on a Thermo-Finnigan ESI LCQ Advantage mass spectrometer (Waltham, MA, USA). LC-ESI-MS analyses were performed on a Phenomenex Jupiter C18 column (5 μm, 150 × 2.0 mm) using a flow rate of 500 μL/min on a ThermoFinnigan Surveyor HPLC system coupled to ESI-MS, using the solvent system: 0.1% TFA in H_2_O (A), 0.1% TFA in MeCN (B).

### Synthesis and purification of compounds 3–5

Peptides were synthesized following the method reported in the preceding paper (Di Cianni et al., 2010). Briefly, the peptides were prepared using the general Fmoc-SPPS strategy on preswelled H-l-Thr(*t*Bu)-ol-2-chloro-trityl resin. Couplings were performed by adding 2 equivalents of protected amino acid activated by HATU and 4 equivalents of NMM in DMF. Each coupling was monitored by the qualitative ninhydrin (Kaiser) test (Kaiser et al., 1970). The cyclization was performed on-resin by second generation Grubbs catalyst (0.5 mol eq. calculated on the basis of 0.5 mmol/g of peptide). After swelling, NH_2_ terminal Fmoc-Agl was deprotected and coupled with Fmoc-d-Phe-OH, affording the on-resin peptides. At this point, all the on-resin peptides were divided in two equal amounts, one portion was deprotected with 20% piperidine in DMF and cleaved [**3a**,**b** with TFA/H_2_O/phenol (96:2:2, 3 h); **4a**,**b**, and **5a**,**b** with TFA/H_2_O/phenol (70:28:2, 2.30 h)]. The aqueous solutions of the peptides **3a**–**5a** were pre-purified by solid phase extraction and after subjected to the purification by semipreparative RP-HPLC and subsequently characterized by ESI-MS. Analytical RP-HPLC and ESI-MS analysis of the crude compounds revealed two chromatographic peaks with the same MW for compounds **3a**–**5a**, corresponding to the geometric isomers (*Z/E* ratio ≈ 90:10). Compounds were then purified by semipreparative RP-HPLC, and the most abundant chromatographic peaks were collected. For all the products, HPLC purity was 97%. The other portion with the deprotected on-resin peptides **3a**–**5a** were coupled with the azamacrocycle DOTA, by adding 2 equivalents of protected DOTA-tris-*t*Bu ester activated by 2 equivalents of HATU and 4 equivalents of NMM in DMF. Also in this case the coupling was monitored by the Kaiser test. The crude peptides **3b**–**5b** were cleaved from the solid support and purified as already described.

### Determination of somatostatin receptor affinity profiles

Determination of Somatostatin receptors affinity was performed at CEREP (Le Bois l'Evêque, B. P. 3,001, 86,600 Celle l'Evescault, France). Cell membrane pellets were prepared from human sst_2_-expressing endogeneous IMR32 cells, and sst_5_-expressing human CHO cells. For each of the tested compounds, complete displacement experiments with the universal SRIF radio-ligand [Leu^8^, D-Trp^22^, ^125^I-Tyr^25^]-SRIF-28 (125I-[LTT]-SRIF-28) (2000 Ci/mmol; Anawa, Wangen, Switzerland) were performed. As control, unlabeled seglitide and SRIF-14 were run in parallel, using the same increasing concentrations, with sst_2_ and sst_5_ subtypes, respectively. *IC*_50_ values were calculated by non-linear regression analysis of the competition curves generated with mean replicate values using Hill equation curve. The analysis was performed using software developed at CEREP (Hill software) and validated by comparison with data generated by the commercial software SigmaPlot 4.0 for Windows.

### HPLC estimation of hydrophobicity

Analytical RP-HPLC was run on a Thermo Finnigan Surveyor HPLC equipped with a Phenomenex aqua C18 column 300 Å 5 μm (250 mm × 4.6 mm). The solvent systems used for gradients were A (0.1% TFA in H_2_O) and B (0.1% TFA in CH_3_CN). The flow rate was 1.0 mL/min, with a linear gradient from 40 to 90% of B in 20 min. The chromatographic peaks were monitored with a PDA detector at 254 nm.

A solution of each compound (1 mg/mL) was prepared in HPLC-grade water and 20 μL of solution were injected into the instrument. Each compound was repeated in triplicate and the average retention time was then calculated.

### NMR spectroscopy

The samples for NMR spectroscopy were prepared by dissolving the appropriate amount of peptide in 0.55 mL of ^1^H_2_O (pH 5), 0.05 ml of ^2^H_2_O to obtain a concentration 1–2 mM of peptides and 200 mM of SDS-d_25_. TSP was used as internal chemical shift standard. The water signal was suppressed by gradient echo (Hwang and Shaka, 1995). NMR experiments were recorded on a Varian Inova-Unity 700 MHz at 308.1 K. 1D ^1^H NMR spectra of the new compounds **3b**–**5b** are reported in Figure S1 of the Supplementary Material. Complete ^1^H NMR chemical shift assignments were effectively achieved for all the analyzed peptides (Tables S3–S5) according to the Wüthrich procedure (Wüthrich, 1986) via the usual systematic application of TOCSY (Braunschweiler and Ernst, 1983) and NOESY (Jeener et al., 1979) experiments recorded in the phase-sensitive mode using the method from States (States et al., 1982).

Typical data block sizes were 2,048 addresses in t_2_ and 512 equidistant *t*_1_ values. Before Fourier transformation, the time domain data matrices were multiplied by shifted sin^2^ functions in both dimensions. A mixing time of 70 ms was used for the TOCSY experiments. NOESY experiments were run with mixing times of 100 and 200 ms. The qualitative and quantitative analyses of TOCSY and NOESY spectra were obtained with the support of the XEASY software package (Bartels et al., 1995).

### Structural determinations and computational modeling

The NOE-based distance restraints were obtained from NOESY spectra collected with the mixing time of 100 ms. The NOE cross peaks were integrated with the XEASY program and were converted into upper distance bounds using the CALIBA program incorporated into the program package DYANA (Güntert et al., 1997). Only NOE derived constraints (Tables S6–S8) were considered in the annealing procedures. In a first calculation run, all the upper distance bounds were used, generating an ensemble of 100 structures with the simulated annealing standard protocol of the program DYANA. For all peptides, a number of consistent (i.e., in all calculated structures) violated upper limit constraints (>0.1 Å) were observed (Tables S6–S8). These violations were discarded in a subsequent MD run. This step was repeated until no violation was observed (two runs were enough for all peptides). Thus, we obtained a first family of structures (family I). In a second MD cycle, the violated upper limit constraints of the first cycle were up-weighted (10-fold) for the contribution to the target energy function of DYANA. Hence, we obtained a new set of violated constraints which were discarded in the subsequent MD runs. After two MD runs, no violations were observed. In the final calculation run, we applied the same weight to the not discarded constraints and obtained a second family of structures (family II). Since, the two sets of violations had no common member we did not repeat further the described procedure.

Finally, 20 structures for each family of peptides **3b**–**5b** were chosen, whose interprotonic distances best fitted NOE derived distances, and then refined through successive steps of restrained and unrestrained energy minimization calculations using the Discover algorithm (Accelrys, San Diego, CA) and the consistent valence force field (CVFF) (Maple et al., 1988).

The minimization lowered the total energy of the structures. The final structures were analyzed using the InsightII program (Accelrys, San Diego, CA). Graphical representations were carried out with the UCSF Chimera package (Pettersen et al., 2004). The root-mean-squared-deviation analysis between energy-minimized structures was carried out with the program MOLMOL (Koradi et al., 1996).

## Results and discussion

### Peptide synthesis and purification

The synthesis of dicarba-analogs followed the procedure described in our previous article (Di Cianni et al., 2010). All the syntheses take advantages from the Solid Phase Peptide Synthesis protocol, allowing to rapidly afford the products in the range from a μM to mM scale and with high purity level.

**Scheme 1:**
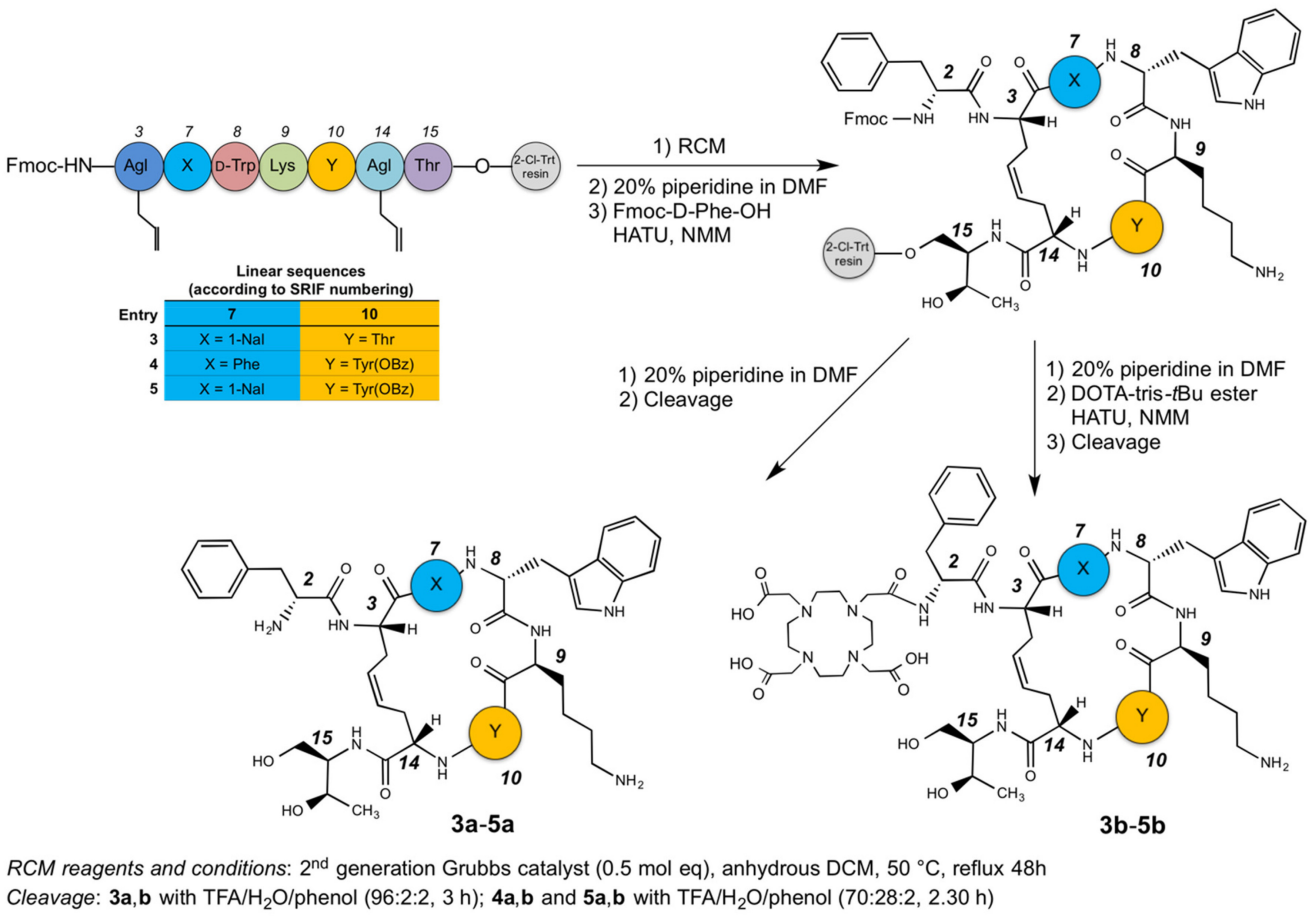
**Synthetic route to compounds 3a-5a and 3b-5b**.

The syntheses of the linear peptides were performed on a 2-chloro-trityl resin pre-loaded with the protected aminoalcool H-l-Thr(*t*Bu)-ol and following the Scheme 1. The peptide chain elongation, according to the selected amino acid sequence, was achieved by the coupling of a pre-activated Fmoc-protected amino acid with HATU and NMM. All the couplings were checked by the Kaiser test (Kaiser et al., 1970). Once the syntheses of the linear peptides were completed, the corresponding dicarba-cyclopeptides were obtained by RCM reactions catalyzed with the second generation Grubb's catalyst in anhydrous DCM for 48 h at 50°C.

At this point, the last amino acid residue d-Phe, was coupled to the three on-resin cyclopeptides and the resins were divided in two halves. One of these was cleaved in order to obtain the free dicarba-cyclopeptide **3a**–**5a**, while the other underwent to the DOTA coupling on the free amino-group of the d-Phe residue with the DOTA-tris-*t*Bu ester, suitably activated with HATU and NMM. Also in this case, the compounds were cleaved from the solid support, affording the crude products **3b**–**5b**. The cleavage of compounds **3a**,**b** was carried out with a mixture of TFA/H_2_O/phenol (96:2:2) for 3 h at r.t., while compounds **4a**,**b**, and **5a**,**b**, that needed some milder conditions since the OBz must be retained on the side-chain of the Tyr residue, were treated with TFA/H_2_O/phenol (70:28:2) for 2.5 h. All compounds obtained were pre-purified by SPE. The concentrated solutions of the raw products were adsorbed on the SPE and eluted with an increased percentage of CH_3_CN in H_2_O (from 0 to 100%). The enriched fractions were then purified by semipreparative RP-HPLC and characterized by ESI-MS (see Tables S1, S2). For each peptide, the HPLC chromatogram showed two peaks with the same MW, corresponding to the geometric isomers (*Z/E* ratio ≈ 90:10). In particular, the *Z* structure of the C-C = C-C tether of the sample, eluted at higher R_*t*_, was recovered and the *cis-*structure ascertained by ^1^H NMR inspection. In particular, the geometry of the double bond of compounds **3b**–**5b** was confirmed as *cis* (*E*) from the coupling constant value (^3^J_CH=CH_ = 11 Hz) between the two olefinic protons of the bridge (Figure S2) and the relative strong NOE between the same olefinic H_γ_s. Since, the signals of the two olefinic protons of all compounds **3b**–**5b** overlapped each other in the NMR spectra acquired in SDS solutions, previous parameters were extracted from spectra acquired in D_2_O solution. The HPLC purity of each compound was >97%, and the isolated structure showed unique *Z* configuration, confirmed by NMR analysis. No oligomeric by-products were observed.

### Binding affinity to sst_2,5_ receptors

The three DOTA conjugated compounds **3b**, **4b**, and **5b** were tested for their ability to bind to the sst_2_ and sst_5_ receptors subtypes in complete displacement experiments using the universal somatostatin radioligand [^125^I]-[Leu^8^,d-Trp^22^,Tyr^25^]-somatostatin-28. *IC*_50_ values were calculated after quantification of the data using a computer-assisted image processing system (Table 1). Receptor subtypes sst_2_ and sst_5_ were chosen for preliminary binding assays because of their overexpression in some tumor types (Miller et al., 1995). In Table 1, the affinities of the parent unconjugated compounds **3a**, **4a**, and **5a** are also reported for comparison (Di Cianni et al., 2010).

Bearing in mind that affinity values >100 nM cannot permit to consider the related compounds of pharmacological interest, from data reported in Table 1 it clearly appears that **3b** maintains a fairly good affinity for sst_2_ while it loses affinity for sst_5_ subtype. The opposite case is reported for the Tyr(OBz)^10^ containing derivative **4b** that shows a moderate reduction of the affinity at the sst_5_ receptor but a total loss of sst_2_ affinity. In the case of compound **5b**, after the conjugation with DOTA there is roughly no effect on sst_2_ activity while the sst_5_ affinity is reduced (2.4-fold). Nevertheless, the binding ability with sst_5_ still remains of significant interest.

### HPLC estimation of hydrophobicity

RP-HPLC retention time (*t*_R_) measurements can give an idea of the difference in hydrophobicity of our peptides after the conjugation with DOTA (Table 2; Tachi et al., 2002; Hossain et al., 2011).

**Table 2:** **RP-HPLC comparison of retention time of dicarba-analogs and their respective DOTA-conjugated analogs**.

**Compound**	***T*_R_ (MIN)**	**Δ *T*_R_ (MIN)**
3a	6.14	0.65
3b	6.79	
4a	10.39	0.55
4b	10.94	
5a	12.02	0.55
5b	12.57	

Compound **3a** is, as expected, the less hydrophobic analog having the –OH group of the Thr^10^ side chain in the place of the lipophilic Tyr(OBz)^10^ residue of **4a** and **5a**. On the other hand, it is not surprisingly that **5a** is still more hydrophobic than **4a** because of the outcome of the aromatic naphtyl side chain in position 7. The introduction of the DOTA moiety at the amino end of these analogs enhanced the affinity of the entire molecule for the stationary phase through a slight increment of the chromatographic retention times, at least in our experimental conditions but did not alter the hydrophobic/hydrophilic nature of the molecules to any significant extent. Noticeably, the contribution to the hydrophobicity carried by the DOTA moiety is almost the same for **3b**–**5b** molecules. Because the lipophilicity = hydrophobicity—polarity (Giaginis and Tsantili-Kakoulidou, 2008), these finds seem to suggest that changes in hydrophobicity and then also in lipophilicity, introduced by the conjugation with the DOTA group, show a very similar trend along the three structures.

### NMR analysis

NMR analysis of the analogs **3b–5b** was performed in SDS-d_25_ micelles solution. The employment of SDS micelles to investigate the conformational properties is justified on the basis of their interaction with a membrane receptor. For peptides that bind membrane receptors, such as GPCR, the use of membrane mimetic solution is suggested, hypothesizing a membrane-assisted mechanism of interactions between the peptides and their receptors (Sargent and Schwyzer, 1986). Hence micelle solutions have been extensively used for conformational studies of peptide hormones and neuropeptides (Grieco et al., 2011; Carotenuto et al., 2013, 2015).

**Figure 3:**
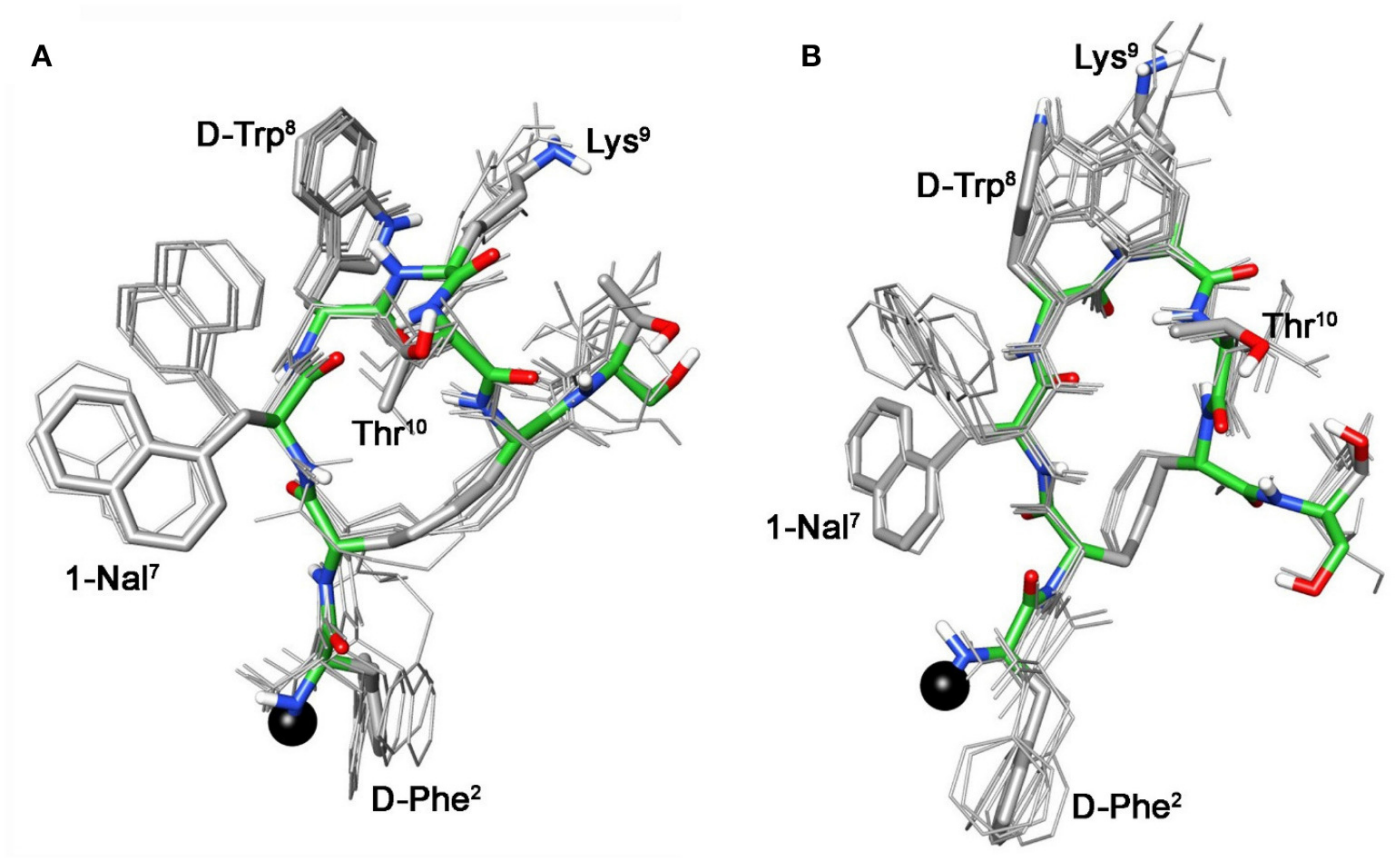
**Superposition of the 10 lowest energy conformers of 3b: family I (A), family II (B)**. Structure models were superimposed using the backbone heavy atoms. Lowest energy conformer is evidenced with thicker stick and different colors of the backbone atoms (carbon, green; nitrogen, blue; oxygen, red; sulfur, yellow). DOTA position is showed as a black ball. Hydrogen atoms of the side chains are hidden for a better view.

For compound **3b**, NMR data resemble those of the corresponding parent **3a** (Table S3) with the main differences located at N-terminal residues 2-3-7 (Note: numbering of the residues follows that of native SRIF, Figure 2). As for **3a** (Di Cianni et al., 2010), NOESY spectra of **3b** showed, simultaneously, both diagnostic connectivities consistent with folded structures and NOE contacts characteristic of extended regions (Table S6). Considering incompatible NOEs separately in different calculation cycles (Di Cianni et al., 2010), two families of conformation were obtained (Figure 3); family I with a type II' β-turn spanning residues d-Trp^8^-Lys^9^, followed by a short 3_10_-helix along residues 10-14-15 (Figure 3A) and family II which differed from the first mainly in that C-terminal residues were in extended conformations (Figure 3B). Compared to the conformations obtained for peptide **3a**, the principal difference in both families is a better definition of d-Phe^2^ side chain which is predominately *gauche-* oriented in **3b**, and a higher conformational freedom for 1-Nal^7^ side chain which populates both *trans* and *gauche-* conformers starting (in **3a**) from a preferred *trans* conformation. d-Phe2 reorientation is probably due to attractive interactions between d-Phe and DOTA moieties, while 1-Nal^7^ movements should be a consequence of the first. Interactions between d-Phe^2^ residue and N-terminal conjugate moieties were already observed in other octreotide dicarba-derivatives (Barragán et al., 2012).

**Figure 4:**
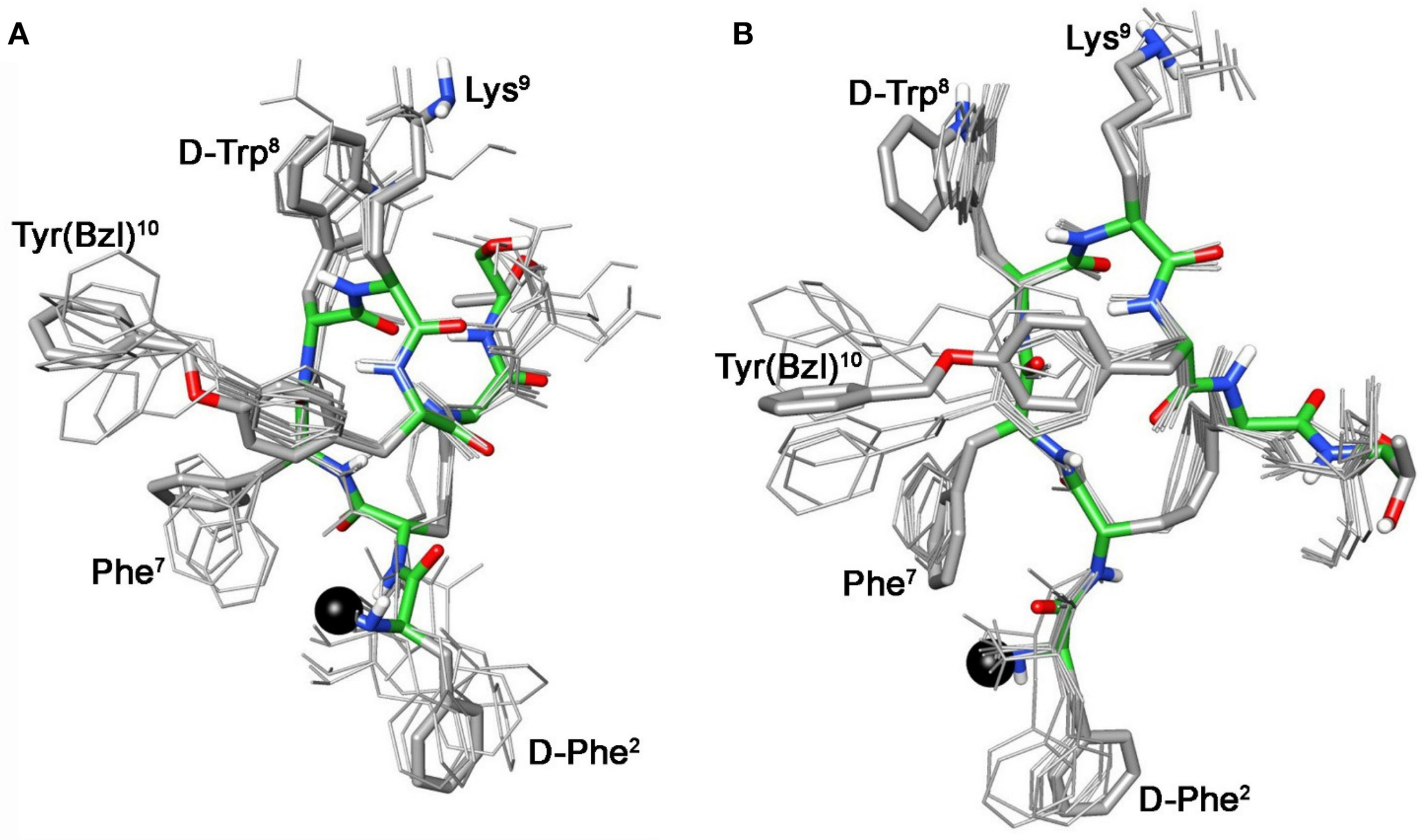
**Superposition of the 10 lowest energy conformers of 4b: family I (A), family II (B)**. Structure models were superimposed using the backbone heavy atoms. Lowest energy conformer is evidenced with thicker stick and different colors of the backbone atoms (carbon, green; nitrogen, blue; oxygen, red; sulfur, yellow). DOTA position is showed as a black ball. Hydrogen atoms of the side chains are hidden for a better view.

**Figure 5:**
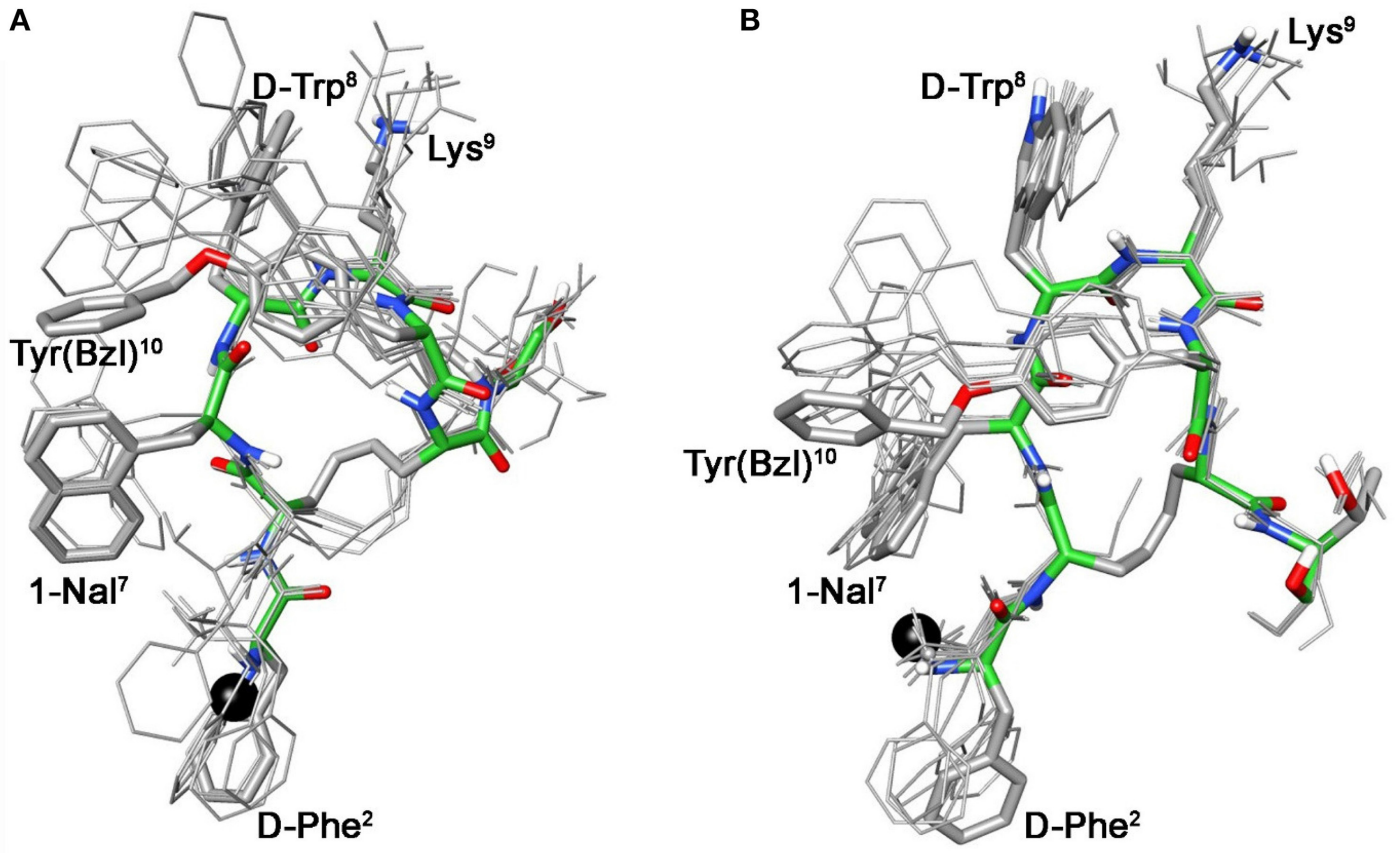
**Superposition of the 10 lowest energy conformers of 5b: family I (A), family II (B)**. Structure models were superimposed using the backbone heavy atoms. Lowest energy conformer is evidenced with thicker stick and different colors of the backbone atoms (carbon, green; nitrogen, blue; oxygen, red; sulfur, yellow). DOTA position is showed as a black ball. Hydrogen atoms of the side chains are hidden for a better view.

These conformational modifications have to account for the loss of activity at receptors, mainly at the subtype sst_5_, because probably the reorientation of the 1-Nal^7^ induces the loss of an important interaction. Differently from NMR spectra of **3a**, those of Tyr(OBz) containing compounds (**4b**, and **5b)** show significant differences regarding all residues compared to the parents **4a** and **5a**, respectively (Tables S4, S5). Some NOE interactions between Tyr(OBz) side chain and both Phe^7^ (1-Nal^7^) and d-Trp^8^ could be observed (Tables S7–S8). Structure calculation explained the experimental NMR data. In fact, apart the *gauche-* side chain orientations of d-Phe^2^ and of Phe^7^ (1-Nal^7^) already described for **3b**, Tyr(OBz) side chain of both extended (family I) and folded (family II) structure clusters of **4b** and **5b** had a *gauche*^+^ orientation (Figures 4, 5). Henceforth, the long Tyr(OBz) side chain is inserted between the aromatic systems of Phe^7^ (1-Nal^7^) and d-Trp^8^. This tight packing blocks the rotation of both Phe^7^ (1-Nal^7^) and d-Trp^8^. As a consequence of this loss of rotational freedom d-Trp^8^ indole moiety is closer, on average, to Lys^9^ residue compared to what happens in the unconjugated peptides; this is in accordance with the significant upfield shifts of all the proton signals of Lys^9^. This compact conformation is favorable to the sst_5_ binding of **5b** (and partially of **4b**) since this compound recovers the affinity toward sst_5_ compared to **3b**.

At the same time, **4b** and **5b** show a moderate/slight reduction of the affinity toward sst_5_ compared to **4a** and **5a**, respectively, again the non-perfect orientation of Phe^7^ (1-Nal^7^) can explain this worsening. Considering the sst_2_ receptor, a slight (**5b**) or total loss (**4b**) of affinity is observed. Unfortunately, due to their poor overall affinities for sst_2_ (both >100 nM), neither compound, **4b** or **5b**, can be considered as a good candidate for eliciting pharmacological activity on this receptor subtype. Consequently, the “cross-arm” orientation of Tyr(OBz) in the context of the β-hairpin structure (Figures 4B, 5B), that is considered the binding conformer at the sst_2_ (Grace et al., 2006), results to be not suitable for sst_2_ receptor.

## Conclusion

In summary, we have prepared three novel DOTA conjugated peptides, which derive from previously developed cyclic dicarba- analogs **3a**, **4a**, and **5a** with low nanomolar affinity toward somatostatin receptors. The new cyclic peptides **3b**, **4b**, and **5b** were tested for their affinity toward sst_2_ and sst_5_ receptors. Compounds **3b** and **5b** maintained moderate to high affinities of their unconjugated parents for the sst_2_ and sst_5_ receptors, respectively. Detailed conformational analysis by solution NMR revealed the possible reasons behind the observed affinity profiles. These very encouraging results will prompt us to load the developed conjugated peptides with different radiometals and to test the novel radiotracers both for diagnostic and therapeutic aims.

## Author contributions

AP, MG, ML, AMP conducted the design and the synthesis. DB, AC, analyzed the NMR data, performed the Molecular Dynamics simulations. MG, EN, AC coordinated the project. AP, MG, AC wrote the main manuscript text. All the authors reviewed the manuscript.

### Conflict of interest statement

The authors declare that the research was conducted in the absence of any commercial or financial relationships that could be construed as a potential conflict of interest.

## Supplementary material

**Analytical data of the synthesized peptides and NMR data of the analyzed peptides**.

## Abbreviations

DOTA: 1,4,7,10-Tetraazacyclododecane-1,4,7,10-tetraacetic acid
DQF-COSY: double quantum filtered correlated spectroscopy
DTPA: 2-[Bis[2-[bis(carboxymethyl)amino]ethyl]amino]aceticacid diethylenetriaminepentaacetic acid
GPCRs: G protein–coupled receptors
HATU: 1-[Bis(dimethylamino)methylene]-1H-1,2,3-triazolo [4,5-b]pyridinium 3-oxid hexafluorophosphate, N-[(Dimethylamino)-1H-1,2,3-triazolo-[4,5-b]pyridin-1-ylmethylene]-N-methylmethanaminium hexafluoro-phosphate N-oxide
HYNIC: 6-[2-(2-sulfonatobenzaldehyde)hydrazono]nicotinyl hydrazinonicotinic acid
NMM: 4-methylmorpholine
NOC: 1-Nal^3^-octreotide
NOESY: nuclear Over-hauser enhancement spectroscopy
RCM: ring closing metathesis
SDS-d_25_: dodecyl-d_25_ sulfate sodium salt
SRIF: somatotropin release–inhibiting factor
TATE: Tyr^3^-octreotate
TOC: [Tyr^3^]octreotide
TOCSY: total correlated spectroscopy
TSP: trimethylsilyl propionate.
