# Supplementary material for: DOTA-Derivatives of Octreotide Dicarba-Analogs with High Affinity for Somatostatin sst2,5 Receptors
Source: Front Chem. 2017 Feb 23;5:8. doi: 10.3389/fchem.2017.00008 (PMC5324734; doi:10.3389/fchem.2017.00008)
Supplement: Supplementary file 1 [file DataSheet1.docx]

Supplementary Material

DOTA-derivatives of octreotide dicarba-analogues with high affinity for somatostatin sst2,5 receptors

Alessandro Pratesi, Mauro Ginanneschi, Marco Lumini, Anna Maria Papini, Ettore Novellino, Diego Brancaccio, Alfonso Carotenuto*

*** Correspondence:** Corresponding Author: [alfonso.carotenuto@unina.](mailto:alfonso.carotenuto@unina.)it

# Supplementary Tables

**Table S1.** RP-HPLC data of the purified analogues **3**-**5**.

| ***Compound*** | ***HPLC method^a^*** | ***R_t_ (min.)^b^*** |
| --- | --- | --- |
| **3a** | 20%-60% B in 20 min. | 13.26 |
| **3b** |  | 15.33 |
| **4a** | 45%-55% B in 20 min. | 9.64 |
| **4b** |  | 12.18 |
| **5a** | 50%-60% B in 10 min. | 7.02 |
| **5b** |  | 10.57 |

*^a^* A: 0.1% TFA in H_2_O; B: 0.1% TFA in CH_3_CN. *^b^* R*_t_* of the pure compounds.

**Table S2.** Mass spectral data of the purified analogues **3**-**5**.

| ***Compound*** | ***[M]^+^ calcd.*** | ***[M+H]^+^ found*** | ***[M+2H]^2+^*** | ***[M+Na]^+^*** |
| --- | --- | --- | --- | --- |
| **3a** | 1031.23 | 1032.0 | 516.46 | 1053.98 |
| **3b** | 1417.63 | 1418.81 | 709.76 | 1440.13 |
| **4a** | 1133.36 | 1134.53 | 567.68 | 1156.66 |
| **4b** | 1519.77 | 1520.54 | 760.54 | 1542,87 |
| **5a** | 1183.42 | 1184,34 | 592,51 | 1205.94 |
| **5b** | 1569.83 | 1570.67 | 785.80 | 1593.23 |

**Table S3.** NMR Resonance Assignments*^a^* of Analogue **3b** in SDS-d_25_ 200mM Solution.

| residue | NH (^3^*J*_αN_, -Δδ/ΔT)^b^ | C^α^H | C^β^H | Others |
| --- | --- | --- | --- | --- |
|  |  |  |  |  |
| D-Phe^2^ | 8.61 | 4.68 | 2.82; 3.16 | 7.31(δ); 7.24(ε) |
| dhDsa-N^c^ | 7.86 (8.0, 7.2) | 4.34 | 2.32; 2.52 | 5.41(γ) |
| 1-Nal^7^ | 8.31 (7.6, 6.2) | 4.75 | 3.43; 3.53 | 7.44 (δ); 8.15, 7.45 (ε); 7.63, 7.67, (ζ); 7.50 7.76 (η) |
| D-Trp^8^ | 7.49 (4.8, 6.6) | 4.20 | 2.42; 2.65 | 6.89(δ); 9.88; 7.10(ε); 7.43, 6.97(ζ); 7.11(η) |
| Lys^9^ | 8.04 (6.2, 4.5) | 3.89 | 1.54; 1.32 | 0.68, 0.41(γ); 1.36(δ); 2.66, 2.71(ε); 7.24(ζ) |
| Thr^10^ | 8.12 (6.0, 2.8) | 4.07 | 4.21 | 1.24(γ) |
| dhDsa-C^c^ | 7.78 (7.6, 6.1) | 4.38 | 2.45; 2.22 | 5.40(γ) |
| Thr(ol)^15^ | 7.32 (7.9, 6.5) | 3.77 | 3.64 | 1.05(γ); 3.51 (ω) |

*^a^*Obtained at 35 °C, pH = 5.5, with TSP (δ 0.00 ppm) as reference shift. Chemical shifts are accurate to ±0.02 ppm. ^b^ ^3^*J*_αN_ coupling constants in Hz. -Δδ/ΔT = temperature coefficients (ppb/K). ^c^ dh-DSA-N: N-terminal portion of dehydrodiaminosuberic acid. dh-DSA-C: C-terminal portion of dehydrodiaminosuberic acid. DOTA signals: 4.10-3.30 ppm.

**Table S4.** NMR Resonance Assignments*^a^* of Analogue **4b** in SDS-d_25_ 200mM Solution.

| residue | NH (^3^*J*_αN_, -Δδ/ΔT)^b^ | C^α^H | C^β^H | Others |
| --- | --- | --- | --- | --- |
|  |  |  |  |  |
| D-Phe^2^ |  | 4.71 | 2.82; 3.21 | 7.25(δ); 7.30(ε) |
| dhDsa-N^c^ | 7.87 (8.1, 6.9) | 4.53 | 2.32; 2.46 | 5.42(γ) |
| Phe^7^ | 7.87 (7.9, 5.6) | 4.75 | 2.99; 3.04 | 7.06(δ); 7.12(ε) |
| D-Trp^8^ | 8.33 (4.7, 6.1) | 4.39 | 2.98; 3.20 | 7.18(δ); 10.01, 7.65(ε); 7.50, 7.14(ζ); 7.13(η) |
| Lys^9^ | 6.95 (5.9, 4.4) | 4.06 | 1.02; 1.38 | 0.42, 0.20(γ); 1.30 (δ); 2.70(ε); 7.30(ζ) |
| Tyr(Bzl)^10^ | 7.97 (6.3, 2.9) | 4.53 | 3.05; 2.93 | 7.12(δ); 6.84(ε);^d^ |
| dhDsa-C^c^ | 8.02 (7.1, 5.3) | 4.54 | 2.39; 2.46 | 5.42(γ) |
| Thr(ol)^15^ | 7.52 (7.8, 5.4) | 3.84 | 3.96 | 1.16(γ); 3.61, 3.73 (ω) |

*^a^*Obtained at 35 °C, pH = 5.5, with TSP (δ 0.00 ppm) as reference shift. Chemical shifts are accurate to ±0.02 ppm. ^b^ ^3^*J*_αN_ coupling constants in Hz. -Δδ/ΔT = temperature coefficients (ppb/K). ^c^ dh-DSA-N: N-terminal portion of dehydrodiaminosuberic acid. dh-DSA-C: C-terminal portion of dehydrodiaminosuberic acid. ^d^ Other signals: CH_2_: 4.98; Arom: 7.37, 7.41, 7.51. DOTA signals: 4.10-3.30 ppm.

**Table S5.** NMR Resonance Assignments*^a^* of Analogue **5b** in SDS-d_25_ 200mM Solution.

| residue | NH (^3^*J*_αN_, -Δδ/ΔT)^b^ | C^α^H | C^β^H | Others |
| --- | --- | --- | --- | --- |
|  |  |  |  |  |
| D-Phe^2^ | 8.33 | 4.69 | 2.87; 3.27 | 7.30(δ); 7.35(ε) |
| dhDsa-N^c^ | 7.80 (7.7, 6.7) | 4.88 | 2.38; 2.50 | 5.47(γ) |
| 1-Nal^7^ | 8.42 (8.4, 5.3) | 4.88 | 3.46; 3.74 | 7.43 (δ); 8.23, 7.38 (ε); 7.63, 7.66, (ζ); 7.54 7.80 (η) |
| D-Trp^8^ | 8.25 (4.8, 6.6) | 3.92 | 2.93; 2.60 | 7.01(δ); 9.96, 7.31(ε); 7.49, 7.12(ζ); 7.11(η) |
| Lys^9^ | 6.08 (6.5, 4.6) | 3.97 | 1.16; 0.65 | 0.03, 0.01(γ); 1.14(δ); 2.60(ε) |
| Tyr(Bzl)^10^ | 7.93 (6.3, 2.7) | 4.59 | 3.04; 2.87 | 7.06(δ); 6.82(ε);^d^ |
| dhDsa-C^c^ | 8.18 (7.0, 5.4) | 4.68 | 2.52; 2.35 | 5.50(γ) |
| Thr(ol)^15^ | 7.62 (7.7, 6.0) | 3.76 | 3.86 | 1.21(γ); 3.63, 3.74 (ω) |

*^a^*Obtained at 35 °C, pH = 5.5, with TSP (δ 0.00 ppm) as reference shift. Chemical shifts are accurate to ±0.02 ppm. ^b^ ^3^*J*_αN_ coupling constants in Hz. -Δδ/ΔT = temperature coefficients (ppb/K). ^c^ dh-DSA-N: N-terminal portion of dehydrodiaminosuberic acid. dh-DSA-C: C-terminal portion of dehydrodiaminosuberic acid. ^d^ Other signals: CH_2_: 4.98, 4.93, Arom: 7.40, 7.50. DOTA signals: 4.10-3.30 ppm.

**Table S6**. NOE Derived Upper Limit Constraints of Compound **3b**

| **Atom1^a^** | **Atom2** | **Upper Limit** | **Violation** | | |
| --- | --- | --- | --- | --- | --- |
|  |  |  | **Helix** | **Extend** | **Ensemble** |
| 2 DPHE HA | 3 DHS HN | 2.44 |  |  |  |
| 2 DPHE HB2 | 3 DHS HN | 3.60 |  |  |  |
| 2 DPHE HB3 | 3 DHS HN | 4.40 |  |  |  |
| 3 DHS HN | 3 DHS HB2 | 3.20 |  |  |  |
| 3 DHS HN | 3 DHS HB3 | 3.20 |  |  |  |
| 3 DHS HN | 3 DHS QB | 3.24 |  |  |  |
| 3 DHS HN | 3 DHS HG | 4.25 |  |  |  |
| 3 DHS HN | 7 NAL HN | 3.82 |  |  |  |
| 3 DHS HA | 3 DHS HG | 3.53 |  | 0.61 |  |
| 3 DHS HA | 14 DHS HG | 4.36 |  |  |  |
| 3 DHS HA | 7 NAL HN | 2.39 |  |  |  |
| 3 DHS QB | 14 DHS HN | 5.72 |  |  |  |
| 3 DHS HG | 7 NAL HN | 5.50 |  |  |  |
| 3 DHS HG | 14 DHS HA | 4.91 |  |  |  |
| 7 NAL HN | 7 NAL HB2 | 2.73 |  |  |  |
| 7 NAL HN | 7 NAL HB3 | 2.73 |  |  |  |
| 7 NAL HN | 7 NAL HD1 | 4.50 |  |  |  |
| 7 NAL HN | 8 DTRP HN | 4.45 |  |  |  |
| 7 NAL HN | 14 DHS HA | 3.81 | 1.58 |  | 0.46 |
| 7 NAL HN | 14 DHS QB | 5.02 |  |  |  |
| 7 NAL HA | 8 DTRP HN | 2.38 |  |  |  |
| 7 NAL HB2 | 7 NAL HD1 | 2.75 |  |  |  |
| 7 NAL HB2 | 7 NAL HD3 | 2.50 |  |  |  |
| 7 NAL HB2 | 8 DTRP HN | 4.13 |  |  |  |
| 7 NAL HB3 | 7 NAL HD1 | 2.75 |  |  |  |
| 7 NAL HB3 | 7 NAL HD3 | 2.50 |  |  |  |
| 7 NAL HB3 | 8 DTRP HN | 4.13 |  |  |  |
| 7 NAL HZ | 8 DTRP HE3 | 5.50 |  |  |  |
| 7 NAL HE3 | 8 DTRP HE3 | 5.50 |  |  |  |
| 7 NAL HD3 | 8 DTRP HN | 4.78 |  |  |  |
| 8 DTRP HN | 8 DTRP HB2 | 2.86 |  |  |  |
| 8 DTRP HN | 8 DTRP HB3 | 2.86 |  |  |  |
| 8 DTRP HN | 8 DTRP QB | 2.56 |  |  |  |
| 8 DTRP HN | 8 DTRP HD1 | 5.50 |  |  |  |
| 8 DTRP HA | 8 DTRP HD1 | 4.81 |  |  |  |
| 8 DTRP HA | 8 DTRP HE3 | 3.06 |  |  |  |
| 8 DTRP HA | 9 LYS HN | 2.45 |  |  |  |
| 8 DTRP HB2 | 9 LYS HN | 4.37 |  |  |  |
| 8 DTRP HB3 | 9 LYS HN | 4.37 |  |  |  |
| 8 DTRP QB | 8 DTRP HE3 | 4.32 |  |  |  |
| 8 DTRP QB | 9 LYS HN | 3.76 |  |  |  |
| 8 DTRP HD1 | 9 LYS HN | 5.50 |  |  |  |
| 8 DTRP HD1 | 9 LYS QG | 6.27 |  |  |  |
| 8 DTRP HE3 | 9 LYS HN | 5.20 |  |  |  |
| 8 DTRP HE3 | 9 LYS HA | 5.37 |  |  |  |
| 8 DTRP HE1 | 9 LYS QG | 6.38 |  |  |  |
| 8 DTRP HE1 | 9 LYS QD | 6.38 |  |  |  |
| 8 DTRP HZ2 | 9 LYS QD | 6.38 |  |  |  |
| 9 LYS HN | 9 LYS HB2 | 3.40 |  |  |  |
| 9 LYS HN | 9 LYS HB3 | 3.40 |  |  |  |
| 9 LYS HN | 9 LYS HG2 | 4.17 |  |  |  |
| 9 LYS HN | 9 LYS HG3 | 4.17 |  |  |  |
| 9 LYS HN | 9 LYS QG | 3.72 |  |  |  |
| 9 LYS HN | 10 THR HN | 2.77 |  |  |  |
| 9 LYS HA | 9 LYS QG | 3.93 |  |  |  |
| 9 LYS HA | 9 LYS QD | 6.35 |  |  |  |
| 9 LYS HA | 10 THR HN | 3.35 |  |  |  |
| 9 LYS HA | 14 DHS HN | 5.50 |  |  |  |
| 9 LYS HA | 15 THO HN | 5.33 |  | 2.90 | 0.41 |
| 9 LYS QB | 10 THR HN | 5.46 |  |  |  |
| 10 THR HN | 10 THR HA | 2.78 |  |  |  |
| 10 THR HN | 10 THR HB | 3.24 |  |  |  |
| 10 THR HN | 14 DHS HN | 2.86 |  | 0.42 |  |
| 10 THR HA | 10 THR HB | 2.85 |  |  |  |
| 10 THR HA | 14 DHS HN | 2.58 | 0.88 |  | 0.31 |
| 10 THR HA | 15 THO HN | 5.30 |  |  |  |
| 10 THR QG2 | 14 DHS HN | 6.53 |  |  |  |
| 14 DHS HN | 14 DHS HB2 | 2.96 |  |  |  |
| 14 DHS HN | 14 DHS HB3 | 2.96 | 0.33 |  |  |
| 14 DHS HN | 14 DHS HG | 4.83 |  |  |  |
| 14 DHS HN | 15 THO HN | 3.25 |  | 1.20 | 0.14 |
| 14 DHS HA | 14 DHS HG | 3.84 |  |  |  |
| 14 DHS HA | 15 THO HN | 2.54 | 0.68 |  |  |
| 14 DHS HB2 | 15 THO HN | 4.20 |  | 0.80 |  |
| 14 DHS HB3 | 15 THO HN | 4.20 |  |  |  |
| 15 THO HN | 15 THO HB | 3.58 |  |  |  |
| 15 THO HA | 15 THO HB | 2.40 |  |  |  |

^a^ 3 DHS: N-terminal portion of dehydrodiaminosuberic acid. 14 DHS: C-terminal portion of dehydrodiaminosuberic acid. NAL: 1-naphtylalanine. THO: Threoninol. ^b^ Violations (Å) observed for the mean structure of the family I (helix), family II (extended) and an ensemble of 20 structures of both families (ensemble).

**Table S7**. NOE Derived Upper Limit Constraints of Compound **4b**

| **Atom1^a^** | **Atom2** | **Upper Limit** | **Violation^b^** | | |
| --- | --- | --- | --- | --- | --- |
|  |  |  | **Helix** | **Extend** | **Ensemble** |
| 2 DPHE HA | 3 DHS HN | 2.63 |  |  |  |
| 2 DPHE HB2 | 3 DHS HN | 3.60 |  |  |  |
| 2 DPHE HB3 | 3 DHS HN | 4.40 |  |  |  |
| 3 DHS HN | 3 DHS HB2 | 3.28 |  |  |  |
| 3 DHS HN | 3 DHS HB3 | 3.28 |  |  |  |
| 3 DHS HN | 3 DHS QB | 3.06 |  |  |  |
| 3 DHS HA | 3 DHS HB2 | 2.73 |  |  |  |
| 3 DHS HA | 3 DHS HB3 | 2.73 |  |  |  |
| 3 DHS HA | 3 DHS QB | 2.47 |  |  |  |
| 3 DHS HA | 3 DHS HG | 3.79 |  |  |  |
| 3 DHS HA | 14 DHS HG | 5.07 |  |  |  |
| 3 DHS HA | 7 PHE QD | 7.51 |  |  |  |
| 7 PHE HN | 7 PHE HB2 | 3.68 |  |  |  |
| 7 PHE HN | 7 PHE HB3 | 3.68 |  |  |  |
| 7 PHE HN | 7 PHE QB | 3.33 |  |  |  |
| 7 PHE HA | 8 DTRP HN | 2.47 |  |  |  |
| 7 PHE QB | 8 DTRP HN | 3.92 |  |  |  |
| 7 PHE QD | 8 DTRP HN | 7.31 |  |  |  |
| 7 PHE QD | 10 TBZ QE | 6.66 |  |  |  |
| 8 DTRP HN | 8 DTRP HB2 | 2.67 |  |  |  |
| 8 DTRP HN | 8 DTRP HB3 | 2.67 |  |  |  |
| 8 DTRP HN | 8 DTRP QB | 2.42 |  |  |  |
| 8 DTRP HN | 8 DTRP HD1 | 5.25 |  |  |  |
| 8 DTRP HA | 8 DTRP HE3 | 2.79 |  |  |  |
| 8 DTRP HA | 9 LYS HN | 2.41 |  |  |  |
| 8 DTRP HA | 10 TBZ HN | 3.82 |  |  |  |
| 8 DTRP HA | 10 TBZ QE | 5.88 |  |  |  |
| 8 DTRP HB2 | 8 DTRP HD1 | 3.71 |  |  |  |
| 8 DTRP HB2 | 8 DTRP HE3 | 4.19 |  |  |  |
| 8 DTRP HB2 | 9 LYS HN | 4.41 |  |  |  |
| 8 DTRP HB3 | 8 DTRP HD1 | 3.71 |  |  |  |
| 8 DTRP HB3 | 8 DTRP HE3 | 4.19 |  |  |  |
| 8 DTRP HB3 | 9 LYS HN | 4.41 |  |  |  |
| 8 DTRP QB | 8 DTRP HD1 | 3.22 |  |  |  |
| 8 DTRP QB | 9 LYS HN | 4.20 |  |  |  |
| 8 DTRP QB | 8 DTRP HE3 | 3.59 |  |  |  |
| 8 DTRP HD1 | 9 LYS QG | 6.41 |  |  |  |
| 8 DTRP HE3 | 9 LYS HN | 4.02 |  |  |  |
| 8 DTRP HE3 | 10 TBZ QE | 7.33 |  |  |  |
| 9 LYS HN | 9 LYS HB2 | 3.10 |  |  |  |
| 9 LYS HN | 9 LYS HB3 | 3.10 |  |  |  |
| 9 LYS HN | 9 LYS QG | 3.51 |  |  |  |
| 9 LYS HN | 10 TBZ HN | 3.03 |  |  |  |
| 9 LYS HA | 9 LYS HG2 | 3.79 |  |  |  |
| 9 LYS HA | 9 LYS HG3 | 3.79 |  |  |  |
| 9 LYS HA | 9 LYS QG | 3.31 |  |  |  |
| 9 LYS HA | 10 TBZ HN | 3.45 |  |  |  |
| 9 LYS HA | 15 THO HN | 5.43 |  | 2.81 | 0.48 |
| 9 LYS HA | 15 THO QG2 | 6.49 |  | 2.13 | 0.36 |
| 9 LYS HB2 | 10 TBZ HN | 3.85 | 0.50 |  |  |
| 9 LYS HB3 | 10 TBZ HN | 3.85 |  |  |  |
| 9 LYS QG | 10 TBZ HN | 6.33 |  |  |  |
| 9 LYS QG | 10 TBZ QD | 6.81 |  |  |  |
| 9 LYS QG | 10 TBZ QE | 6.90 |  |  |  |
| 9 LYS QD | 10 TBZ QD | 7.11 |  |  |  |
| 9 LYS QD | 10 TBZ QE | 7.16 |  |  |  |
| 10 TBZ HN | 10 TBZ HB2 | 2.81 |  |  |  |
| 10 TBZ HN | 10 TBZ HB3 | 3.16 |  |  |  |
| 10 TBZ HN | 14 DHS HN | 2.92 |  | 0.39 |  |
| 10 TBZ HB2 | 14 DHS HN | 3.99 |  | 0.42 |  |
| 10 TBZ HB3 | 14 DHS HN | 3.84 |  | 0.60 |  |
| 10 TBZ QD | 14 DHS HN | 6.67 |  |  |  |
| 10 TBZ QD | 15 THO QG2 | 7.25 | 0.54 |  |  |
| 14 DHS HN | 14 DHS HB2 | 2.55 |  |  |  |
| 14 DHS HN | 14 DHS HB3 | 3.31 | 0.41 |  |  |
| 14 DHS HN | 14 DHS HG | 5.04 |  |  |  |
| 14 DHS HN | 15 THO HN | 3.05 |  | 1.26 | 0.25 |
| 14 DHS HA | 14 DHS HG | 3.73 |  |  |  |
| 14 DHS HA | 15 THO HN | 2.66 | 0.59 |  |  |
| 14 DHS HB2 | 15 THO HN | 3.73 |  | 0.86 |  |
| 14 DHS HB3 | 15 THO HN | 4.38 |  |  |  |
| 15 THO HN | 15 THO HB | 3.67 |  |  |  |
| 15 THO HN | 15 THO QG2 | 4.58 |  |  |  |
| 15 THO HA | 15 THO HB | 2.51 |  |  |  |

^a^ 3 DHS: N-terminal portion of dehydrodiaminosuberic acid. 14 DHS: C-terminal portion of dehydrodiaminosuberic acid. TBZ: Tyr(Bzl). THO: Threoninol. ^b^ Violations (Å) observed for the mean structure of the family I (helix), family II (extended) and an ensemble of 20 structures of both families (ensemble).

**Table S8**. NOE Derived Upper Limit Constraints of Compound **5b**

| **Atom1^a^** | **Atom2** | **Upper Limit** | **Violation** | | |
| --- | --- | --- | --- | --- | --- |
|  |  |  | **Helix** | **Extend** | **Ensemble** |
| 2 DPHE HA | 3 DHS HN | 2.52 |  |  |  |
| 2 DPHE HB2 | 3 DHS HN | 3.62 |  |  |  |
| 2 DPHE HB3 | 3 DHS HN | 4.42 |  |  |  |
| 3 DHS HN | 3 DHS HB2 | 3.27 |  |  |  |
| 3 DHS HN | 3 DHS HB3 | 3.27 |  |  |  |
| 3 DHS HN | 3 DHS QB | 3.05 |  |  |  |
| 3 DHS HA | 7 NAL HN | 2.73 |  |  |  |
| 3 DHS HG | 7 NAL HN | 5.50 |  |  |  |
| 7 NAL HN | 7 NAL HB2 | 3.39 |  |  |  |
| 7 NAL HN | 7 NAL HB3 | 3.39 |  |  |  |
| 7 NAL HA | 7 NAL HD1 | 3.96 |  |  |  |
| 7 NAL HA | 8 DTRP HN | 2.52 |  |  |  |
| 7 NAL HB2 | 7 NAL HD1 | 3.09 |  |  |  |
| 7 NAL HB3 | 7 NAL HD1 | 3.09 |  |  |  |
| 7 NAL HB2 | 7 NAL HD3 | 3.70 |  |  |  |
| 7 NAL HB3 | 7 NAL HD3 | 3.70 |  |  |  |
| 7 NAL HD1 | 10 TBZ QE | 6.59 |  |  |  |
| 7 NAL HD3 | 10 TBZ QE | 6.39 |  |  |  |
| 8 DTRP HN | 8 DTRP HB2 | 2.88 |  |  |  |
| 8 DTRP HN | 8 DTRP HB3 | 2.88 |  |  |  |
| 8 DTRP HN | 8 DTRP QB | 2.49 |  |  |  |
| 8 DTRP HN | 8 DTRP HD1 | 5.16 |  |  |  |
| 8 DTRP HA | 8 DTRP HD1 | 4.95 |  |  |  |
| 8 DTRP HA | 8 DTRP HE3 | 2.75 |  |  |  |
| 8 DTRP HA | 9 LYS HN | 2.59 |  |  |  |
| 8 DTRP HA | 10 TBZ HN | 3.77 |  |  |  |
| 8 DTRP HA | 10 TBZ QD | 7.34 |  |  |  |
| 8 DTRP HA | 10 TBZ QE | 7.63 |  |  |  |
| 8 DTRP HB2 | 8 DTRP HD1 | 3.46 |  |  |  |
| 8 DTRP HB2 | 8 DTRP HE3 | 4.08 |  |  |  |
| 8 DTRP HB3 | 8 DTRP HD1 | 3.46 |  |  |  |
| 8 DTRP HB3 | 8 DTRP HE3 | 4.08 |  |  |  |
| 8 DTRP QB | 8 DTRP HD1 | 3.26 |  |  |  |
| 8 DTRP HD1 | 9 LYS HG2 | 5.26 |  |  |  |
| 8 DTRP HD1 | 9 LYS HG3 | 5.26 |  |  |  |
| 8 DTRP HD1 | 9 LYS QG | 4.93 |  |  |  |
| 8 DTRP HE3 | 9 LYS HN | 4.11 |  |  |  |
| 8 DTRP HE3 | 9 LYS QG | 6.37 |  |  |  |
| 8 DTRP HE3 | 10 TBZ QE | 7.43 |  |  |  |
| 8 DTRP HE1 | 9 LYS QD | 6.37 |  |  |  |
| 8 DTRP HE1 | 9 LYS QE | 6.58 |  |  |  |
| 9 LYS HN | 9 LYS HB2 | 2.71 |  |  |  |
| 9 LYS HN | 9 LYS HB3 | 2.71 |  |  |  |
| 9 LYS HN | 9 LYS HG2 | 4.16 |  |  |  |
| 9 LYS HN | 9 LYS HG3 | 4.16 |  |  |  |
| 9 LYS HN | 9 LYS QG | 3.86 |  |  |  |
| 9 LYS HN | 10 TBZ HN | 2.94 |  |  |  |
| 9 LYS HN | 10 TBZ QD | 7.59 |  |  |  |
| 9 LYS HA | 9 LYS QG | 3.91 |  |  |  |
| 9 LYS HA | 9 LYS QD | 5.40 |  |  |  |
| 9 LYS HA | 10 TBZ HN | 3.47 |  |  |  |
| 9 LYS HA | 14 DHS HN | 4.99 |  |  |  |
| 9 LYS HA | 15 THO HN | 5.51 |  | 2.76 | 0.48 |
| 9 LYS HA | 15 THO QG2 | 6.48 |  | 2.10 | 0.35 |
| 9 LYS HB2 | 10 TBZ HN | 3.80 | 0.50 |  |  |
| 9 LYS HB3 | 10 TBZ HN | 3.80 | 0.50 |  |  |
| 9 LYS QB | 10 TBZ QD | 8.49 |  |  |  |
| 9 LYS QG | 10 TBZ QE | 7.58 |  |  |  |
| 10 TBZ HN | 10 TBZ HB2 | 3.62 |  |  |  |
| 10 TBZ HN | 10 TBZ HB3 | 3.62 |  |  |  |
| 10 TBZ HN | 10 TBZ QE | 7.58 |  |  |  |
| 10 TBZ HN | 14 DHS HN | 3.01 |  | 0.33 |  |
| 10 TBZ HA | 10 TBZ HB2 | 2.88 |  |  |  |
| 10 TBZ HA | 10 TBZ HB3 | 2.88 |  |  |  |
| 10 TBZ HA | 14 DHS HN | 2.64 | 0.84 |  | 0.24 |
| 10 TBZ HB2 | 14 DHS HN | 3.96 |  | 0.45 |  |
| 10 TBZ HB3 | 14 DHS HN | 3.96 |  | 0.61 |  |
| 14 DHS HN | 14 DHS HB2 | 2.98 |  |  |  |
| 14 DHS HN | 14 DHS HB3 | 2.98 |  |  |  |
| 14 DHS HN | 14 DHS HG | 4.48 |  |  |  |
| 14 DHS HN | 15 THO HN | 3.19 |  | 1.23 | 0.15 |
| 14 DHS HA | 14 DHS HB2 | 2.96 |  |  |  |
| 14 DHS HA | 14 DHS HB3 | 2.96 |  |  |  |
| 14 DHS HA | 14 DHS HG | 3.78 |  |  |  |
| 14 DHS HA | 15 THO HN | 2.63 | 0.61 |  |  |
| 14 DHS HB2 | 15 THO HN | 3.84 |  | 0.77 |  |
| 14 DHS HB3 | 15 THO HN | 4.38 |  |  |  |
| 15 THO HN | 15 THO HB | 3.61 |  |  |  |
| 15 THO HN | 15 THO QG2 | 4.87 |  |  |  |
| 15 THO HA | 15 THO HB | 2.45 |  |  |  |

^a^ 3 DHS: N-terminal portion of dehydrodiaminosuberic acid. 14 DHS: C-terminal portion of dehydrodiaminosuberic acid. NAL: 1-naphtylalanine. TBZ: Tyr(Bzl). THO: Threoninol. ^b^ Violations (Å) observed for the mean structure of the family I (helix), family II (extended) and an ensemble of 20 structures of both families (ensemble).

# Supplementary Figures


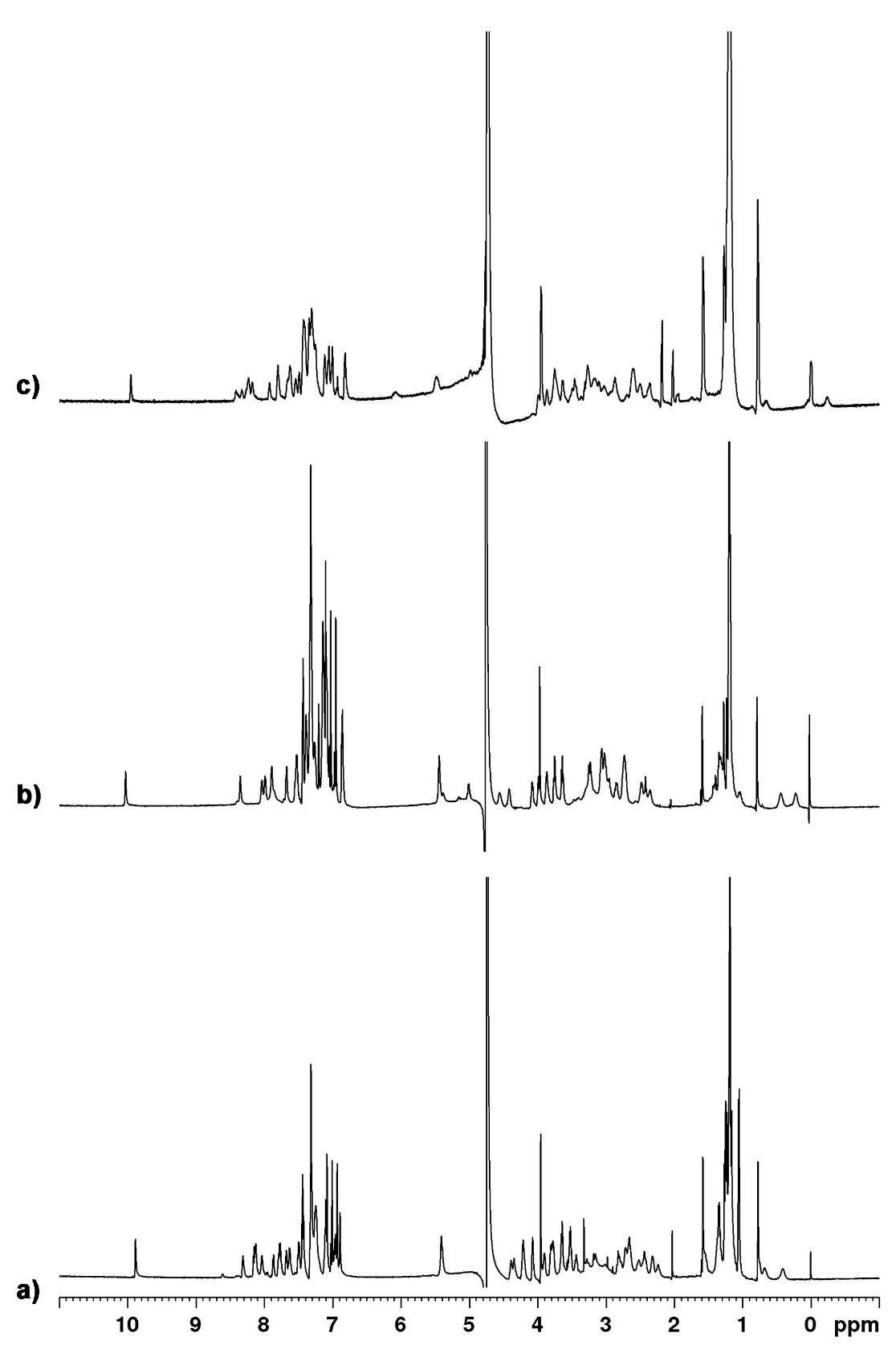


**Supplementary Figure S1.** ^1^H NMR spectra of compounds **3b**(a) **4b** (b) and **5b** (c) acquired in 200 mM SDS-d_25_ solution.


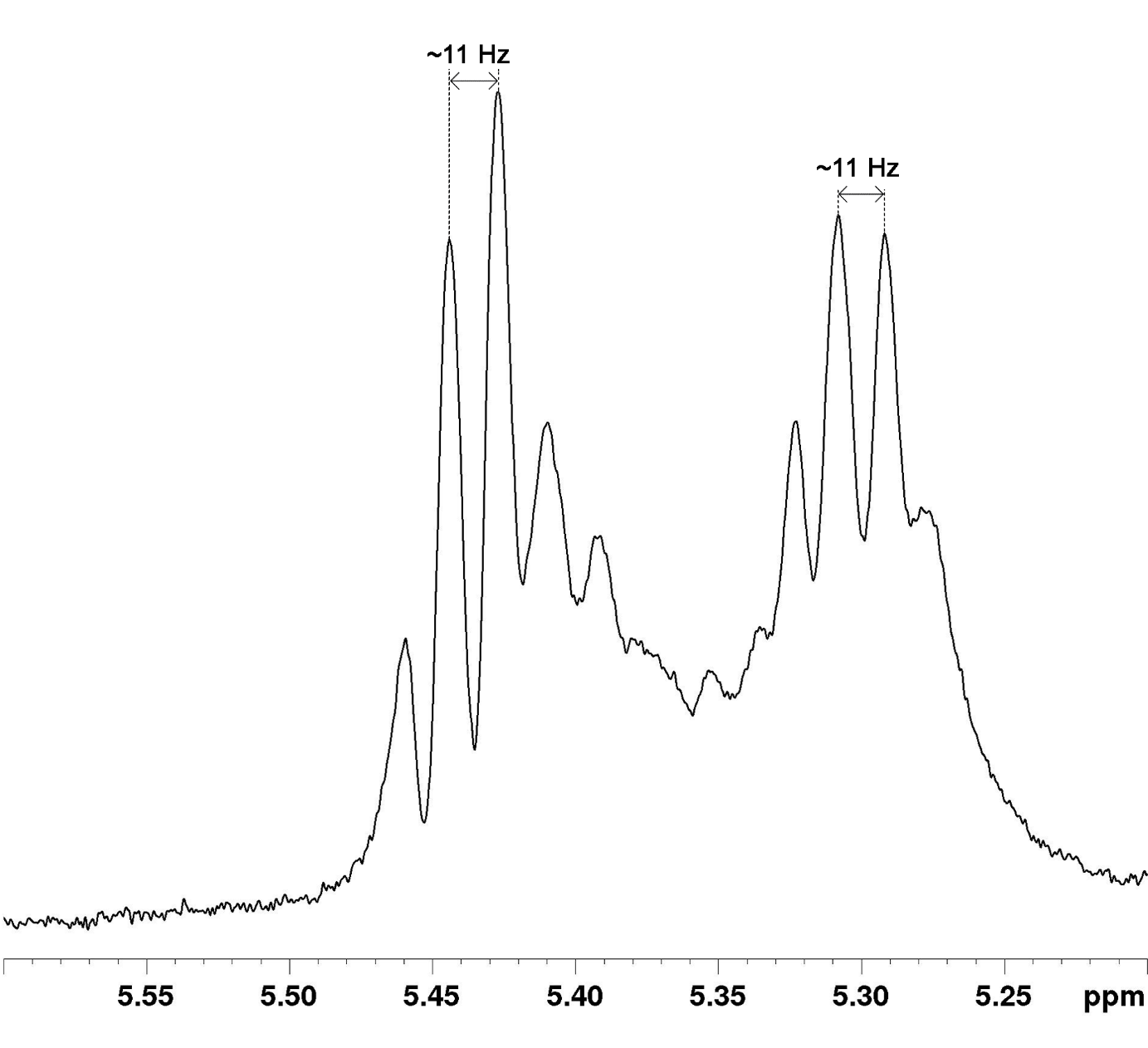


**Supplementary Figure S2.** Particular of the ^1^H NMR spectrum of compound **4b** acquired in D_2_O showing the olefinic proton signals and the relevant coupling constant (doublet of doublet of doublets with three almost equal splittings). Compounds **3b** and **5b** show very similar spectra.
